# Predicting and mitigating fatigue effects due to sleep deprivation: A review

**DOI:** 10.3389/fnins.2022.930280

**Published:** 2022-08-05

**Authors:** Kylie C. Kayser, Vannia A. Puig, Justin R. Estepp

**Affiliations:** ^1^Air Force Research Laboratory, Oak Ridge Institute for Science and Education, Wright-Patterson AFB, OH, United States; ^2^711th Human Performance Wing, Air Force Research Laboratory, Wright-Patterson AFB, OH, United States

**Keywords:** fatigue, non-invasive brain stimulation, fatigue mitigation, sleep deprivation, task performance, physiological effects, cognitive functioning

## Abstract

The deleterious effects of insufficient sleep have been well-established in the literature and can lead to a wide range of adverse health outcomes. Some of the most replicated findings demonstrate significant declines in cognitive functions such as vigilance and executive attention, psychomotor and cognitive speed, and working memory. Consequently, these decrements often lead individuals who are in a fatigued state to engage in substandard performance on everyday tasks. In the interest of curtailing these effects, prior work has attempted to identify mechanisms that predict fatigue onset and develop techniques to mitigate its negative consequences. Nonetheless, these results are often confounded by variables such as an individual’s resistance to fatigue, sleep history, and unclear distinctions about whether certain performance decrements are present due to fatigue or due to other confounding factors. Similar areas of research have provided approaches to produce models for the prediction of cognitive performance decrements due to fatigue through the use of multi-modal recording and analysis of fatigue-related responses. Namely, gathering and combining response information from multiple sources (i.e., physiological and behavioral) at multiple timescales may provide a more comprehensive representation of what constitutes fatigue onset in the individual. Therefore, the purpose of this review is to discuss the relevant literature on the topic of fatigue-related performance effects with a special emphasis on a variety of physiological and behavioral response variables that have shown to be sensitive to changes in fatigue. Furthermore, an increasing reliance on sleep loss, meant to assist in meeting the demands of modern society, has led to an upsurge in the relevance of identifying dependable countermeasures for fatigued states. As such, we will also review methods for the mitigation of performance effects due to fatigue and discuss their usefulness in regulating these effects. In sum, this review aims to inspire future work that will create opportunities to detect fatigue and mitigate its effects prior to the onset of cognitive impairments.

## Introduction

Cognitive performance is affected by sleep loss; well-replicated effects include slower response times (Ogilvie and Wilkinson, 1984; Philip et al., 2004; Wesensten et al., 2005; Jackson et al., 2016; Patrick et al., 2017; Boardman et al., 2018), increased lapses in attention (Durmer and Dinges, 2005; Banks and Dinges, 2007; Lim and Dinges, 2008; Goel et al., 2009; Anderson and Dickinson, 2010; Banks et al., 2010; Jackson et al., 2016; Lo et al., 2016), and declines in memory performance (Dodds et al., 2011; Lo et al., 2016; Patrick et al., 2017; Boardman et al., 2018; Deliens et al., 2018; McMahon et al., 2018; Gerhardsson et al., 2019). Some of these changes have been modeled using computational representations of cognitive processes, as well. For instance, Gunzelmann et al. (2009) modeled the cognitive effects of fatigue on vigilant attention. This model describes how fatigue affects cognitive processes by reducing the usefulness of potential behaviors when responding to a certain informational input. In turn, this reduction causes the behaviors to fall below an action threshold (Walsh et al., 2017). In other words, increasing time awake decreases the likelihood of engaging the appropriate response to a particular input.

Prior work has also shown the effects of fatigue on behavioral and subjective outcomes. For example, sleep-deprived individuals are more likely to engage in risk-taking behaviors (Killgore et al., 2011), have less inhibitory control (Chuah et al., 2006), and are more likely to make mistakes (i.e., drivers and pilots; Previc et al., 2009; Jackson et al., 2016). Moreover, the ability to detect and correct errors is hampered by increased wakefulness (Hsieh et al., 2007). Research has also demonstrated that performance decrements associated with fatigue are coupled with increases in subjective sleepiness (Philip et al., 2012; Paech et al., 2016) and decreases in motivation and mood state (Pigeau et al., 1995; Chuah et al., 2006; Franzen et al., 2008).

When discussing fatigue, it is important to distinguish between fatigue due to sleep deprivation and fatigue due to sleep restriction. Sleep loss due to sleep deprivation is characterized as a period of prolonged wakefulness (i.e., pulling an all-nighter; Reynolds and Banks, 2010), whereas sleep restriction is defined as a chronic reduction of sleep time (i.e., 2–3 h of sleep for 5 days in a row; Banks and Dinges, 2007). Sleep deprivation is more impactful in the short term as its effects present themselves after only one night of continuous sleep loss (Hsieh et al., 2007; Tobaldini et al., 2013; Patrick et al., 2017) and can increase in severity with extended wakefulness (Pigeau et al., 1995; Previc et al., 2009; Short and Banks, 2014). Nonetheless, the long-term effects of sleep restriction are known to mimic those of sleep deprivation (Axelsson et al., 2008; Goel et al., 2009; Philip et al., 2012). In real-life settings, sleep restriction is more common as a result of medical conditions, sleep disorders, and lifestyle (Banks and Dinges, 2007); whereas periods of sleep deprivation are most often found in occupations such as healthcare, aviation, mining, transportation, and military settings where work hours extend to all hours of the day (Short and Banks, 2014; Paech et al., 2016). However, in laboratory settings, sleep deprivation is more commonly studied since less time is required for fatigue responses to be observed (Belenky et al., 2003). Indeed, research has shown that the number of adults getting an inadequate amount of sleep per night has increased over the past several decades (Ford et al., 2015; Watson et al., 2015), with more time being spent on activities such as work and socialization (Basner et al., 2007).

Consequently, it is commonplace for individuals to seek interventions that reduce fatigue. Caffeine is one of the most common substances used to counter the effects of sleep loss as it is ubiquitously found in consumer goods, over-the-counter medications, and supplements. It is often used in research for its notable mitigating properties (Wesensten et al., 2005; Killgore et al., 2011; Killgore and Kamimori, 2020a). Prescription medications, such as modafinil, are also used to treat sleep disorders and have been studied as a fatigue countermeasure (Pigeau et al., 1995; Wesensten et al., 2005). More recently, non-invasive nervous system modulation through both the brain [i.e., transcranial direct current stimulation (tDCS); McIntire et al., 2014, 2017] and the periphery (i.e., vagal nerve stimulation; Rizzo et al., 2003; McIntire et al., 2021) have also been studied.

*In situ*, and without complete and accurate data about an individual’s sleep history, the prediction of performance decrements due to fatigue is difficult. In circumstances such as driving a vehicle or flying an aircraft, real-time behavioral metrics, i.e., from eye-tracking software, may be useful to predict performance outcomes, but are not uniquely indicative of fatigue. Changes in these metrics can also correlate to changes in different states thus making it difficult to ascertain the specific cause and to uniquely identify a single factor as the underlying reason. For example, blink frequency increases as fatigue increases (Morris and Miller, 1996), but an increase in blink frequency is also correlated to a decrease in cognitive load (Svensson and Wilson, 2002) and a decrease in the complexity of a driving environment (Faure et al., 2016); therefore, by only using frequency of blinks, it is nearly impossible to discern whether the increase in frequency was solely caused by fatigue. Furthermore, fatigue often causes an increase in attentional lapses (Jackson et al., 2016), but attentional lapses are not uniquely predictive of fatigue since it can also result from a higher cognitive load (Buckley et al., 2016), mind-wandering (Chernyshev et al., 2015), and boredom (Cheyne et al., 2006). Therefore, the inability to determine the distinct cause for the change in behavioral metrics makes it impossible to claim that the change is uniquely predictive of fatigue. Changes in performance may also be due to distraction (Nabatilan et al., 2012), alcohol impairment (Shiferaw et al., 2014), time-on-task (Cazzoli et al., 2014), or habituation (Van Dongen et al., 2003a; Philip et al., 2012). Results obtained from these metrics can also be influenced by boredom and practice effects, though some work has made efforts to establish sensitivity levels as a means to diminish confounding interpretations (Dinges et al., 1997; Russo et al., 2003).

A source of information that remains underexplored is a user’s physiological response to performing under fatigue. Prior work in the transportation industry has explored the use of eye tracking technology to assess fatigue. For instance, Jackson et al. (2016) observed an increase in eyelid closure and number of attentional lapses following a period of sleep deprivation. Moreover, it has been found that oculomotor measures, such as saccadic velocity, pupil diameter, and latency to pupil constriction, are affected by sleep deprivation (Russo et al., 2003; Rowland et al., 2005). In the extant literature, many other sources of physiological response data from both the brain and autonomic nervous system have been explored. One such physiological response is the effect of sleep loss on the sympathetic and parasympathetic nervous systems. Research in this area has evidenced that sleep loss leads to increases in sympathetic activity and decreases in parasympathetic activity (Kato et al., 2000; Dettoni et al., 2012). Differences in automatic thermoregulation have also been identified. For example, Vaara et al. (2009) found that the disruption of circadian rhythms, resulting from fatigue, leads to reduced activity in certain regulatory brain regions (i.e., the hypothalamus), which causes a cumulative decrease of body temperature over the period of sleep deprivation.

In similar areas of study, it has been evidenced that multimodal recording and analysis of physiological response data produce more accurate models for the prediction of cognitive performance decrements (Estepp and Christensen, 2015). These multi-modal data source architectures combine information from multiple physiological sensors at multiple functional timescales. It is likely that the prediction of fatigue-related performance effects is best served by observing multiple sources of physiological and behavioral response information. Herein, we review the relevant literature on this topic with a special emphasis on a variety of physiological and behavioral response variables that have shown to be sensitive to changes in fatigue. Simply predicting fatigue is not sufficient to elucidate performance enhancement; therefore, we also review methods for the mitigation of fatigue-related performance effects. In an ideal, end-user product, prediction of fatigue would facilitate an individualized mitigation strategy specific to the environment and desired outcome of the task at hand. It is our hope that this review presents a consolidated perspective on the most viable approaches for the prediction and mitigation of fatigue-related performance effects such that future work on the design and evaluation of closed-loop systems with those aims be well-served.

## Sleep deprivation vs. sleep restriction

Sleep loss, regardless of originating from sleep deprivation or sleep restriction, has widespread neurological, behavioral, and physiological consequences. Past research has suggested that different kinds of sleep loss are represented by their own distinct characteristics as well as some notable similarities. As the main purpose of this review is to address fatigue-related performance effects due to sleep deprivation, we will dedicate the following section to comparing and contrasting the conceptualization of sleep deprivation and sleep restriction, as well as their distinctive impacts on human functioning. Subsequently, in the sections that follow, the term “fatigue” will be only used to describe the effects of sleep deprivation on performance, unless otherwise specified.

Sleep deprivation is defined as a complete absence of sleep for at least one night (Reynolds and Banks, 2010). It has been extensively studied since its first empirical investigation in the late nineteenth century (Patrick and Gilbert, 1896) and remains the most common form of sleep loss that has been explored in laboratory settings, particularly because sleep deprivation experiments often produce large behavioral deficits in less time when compared to sleep restriction experiments (Belenky et al., 2003). As such, the effects of sleep deprivation on human performance are well-established and have been numerously replicated. Sleep restriction is defined as a reduced duration of sleep for multiple, consecutive nights and is considered the most common form of sleep loss experienced in everyday settings (Banks and Dinges, 2007). In fact, the American Academy of Sleep Medicine and Sleep Research Society recommends engaging in at least 7 h of sleep per night in order to promote optimal human health (Watson et al., 2015); nonetheless, it is common for individuals to restrict their sleep time as a way to keep up with the demands of modern society. Regardless of its prevalence, early sleep restriction studies produced scarce results mainly due to methodological limitations (Short and Banks, 2014). However, through more rigorous control of its empirical study, sleep restriction has garnered increased attention from the scientific community over the last 20 years, making its effects on human performance presently well-known and documented.

Typical sleep restriction experiments are performed in a laboratory environment. Participants are randomly assigned to one of several different sleep-dose conditions (i.e., 4, 6, or 8 h spent in bed per night) which are kept constant for a number of consecutive nights (Belenky et al., 2003; Van Dongen et al., 2003b). Throughout the experimental session, participants undergo different tests to determine objective and subjective sleepiness, as well as the effects of restricted sleep on performance. Research has shown that a wide range of functions are affected by restricting sleep. For instance, spending less than 6 h in bed per night for several consecutive nights causes an immediate increase in objective and subjective sleepiness (Philip et al., 2012; Short and Banks, 2014). Effects may span across cognitive, physiological, and behavioral aspects. For instance, cognitively, sleep restriction leads to decreases in psychomotor vigilance response speed (Belenky et al., 2003), increases in attentional lapses (Belenky et al., 2003; Axelsson et al., 2008; Mollicone et al., 2010), and increases in false memory formation (Lo et al., 2016). Physiologically, sleep restriction may lead to changes in oculomotor responses (Russo et al., 2003; Wilkinson et al., 2013), alterations in sleep stage duration (Carskadon and Dement, 1981), and increases in sympathetic nervous system activity (Dettoni et al., 2012; Słomko et al., 2018). These deficits often occur in a sleep-dose dependent manner (Belenky et al., 2003; Van Dongen et al., 2003b; Mollicone et al., 2010), signifying that the intensity of such effects may vary based on the amount of sleep loss that has taken place. In contrast, other studies have shown that levels of subjective sleepiness increase rapidly at first but stabilize after a few nights of sleep restriction (Carskadon and Dement, 1981; Philip et al., 2012). For instance, Van Dongen et al. (2003b) found that participants restricted to 4 and 6 h of sleep time initially reported increasing levels of subjective sleepiness but reported only minor changes in these ratings as sleep loss accumulated. In fact, even as their objective performance decreased, their feelings of sleepiness did not reach levels equal to those after 2 nights of sleep deprivation. Therefore, evidence suggests that subjective measurements of sleep are not reliable indicators of performance ability due to sleep restriction, as people are lulled into believing that the consequences of lack of sleep on cognitive function are benign (Van Dongen et al., 2003b; Mollicone et al., 2010; Reynolds and Banks, 2010).

Though different in many aspects, there are effects of sleep restriction that overlap with those found in sleep deprivation conditions. In fact, multiple days of sleep restriction can produce cognitive decrements comparable to 1–2 days of sleep deprivation (Banks and Dinges, 2007). For instance, Van Dongen et al. (2003b) identified performance on the Psychomotor Vigilance Task (PVT) and Digit Symbol Substitution Task (DSST) reaching levels comparable to those in sleep deprivation studies. Prior research has also identified increases in subjective and objective sleepiness, deficits in vigilance, decrements in attention and cognition, decline in mood, changes in glucose metabolism, and changes in heart rate variability as common deficits between these two types of sleep loss (Short and Banks, 2014). Furthermore, it has been observed that both sleep restriction and deprivation are sensitive to the wake-maintenance zone, meaning they both follow the circadian phase where alertness increases and sleepiness decreases during the afternoon (Mollicone et al., 2010; McMahon et al., 2018).

In contrast, one of the most significant differences between the two types of sleep loss is often found in the time-duration and intensity of their effects (Short and Banks, 2014), which are most evident during periods of recovery following sleep loss. For instance, Philip et al. (2012) found that one night of recovery is enough to restore cognitive performance and sleepiness levels following a period of sleep deprivation, whereas it takes multiple nights of recovery sleep after sleep restriction to return performance levels to baseline (Belenky et al., 2003; Axelsson et al., 2008; Banks et al., 2010). Some have posited that the accumulation of sleep loss experienced during multiple nights of sleep restriction causes the brain to undergo adaptive changes to stabilize performance; this adaptive response is thought to prevent the rapid recovery of performance to baseline levels that is often seen after sleep deprivation (Belenky et al., 2003; Axelsson et al., 2008). Another notable difference is found within physiological effects. Słomko et al. (2018) found that subjects in the sleep deprivation condition had higher core body temperatures compared to subjects in the sleep restriction condition. They also found that sleep restriction has a greater effect on the autonomic nervous system than sleep deprivation. Namely, sleep restriction leads to an increase in sympathetic nervous system activity, whereas sleep deprivation does not (Dettoni et al., 2012; Słomko et al., 2018).

Beyond their differences, it has been well-established that sleep deprivation and sleep restriction share a multitude of similar effects; namely, increased sleepiness, deficits in vigilance, attention, cognition, changes in mood state, and changes in heart rate (Short and Banks, 2014). Due to these similarities, and the efficiency of sleep deprivation paradigms, sleep deprivation is often used as a proxy for sleep restriction, as it is not always feasible to keep a participant in the laboratory for multiple consecutive nights. Consequently, there is an overabundance of literature dedicated to sleep deprivation in comparison to sleep restriction. It is because of the aforementioned similarities, as well as its empirical prevalence, that we have chosen to dedicate the following sections to a thorough discussion of fatigue-related effects and mitigations due to sleep deprivation.

## Effects of fatigue

### Fatigue and cognition

The need for sleep is a biological imperative modulated by the suprachiasmatic nuclei of the hypothalamus (Goel et al., 2009). This region also modulates the stability of waking cognitive functions, which declines as the drive for sleep increases (Short and Banks, 2014). During extended wakefulness, an individual’s readiness for sleep increases, resulting in shorter time periods between wake and sleep states (i.e., latencies; Carskadon and Dement, 1987). In turn, this increasing need for sleep is believed to intrude in one’s ability to perform cognitively demanding tasks (Durmer and Dinges, 2005). The effects of sleep deprivation on cognition have been extensively studied in the literature (see Supplementary Table 1; Van Dongen et al., 2003a,b; Durmer and Dinges, 2005; Franzen et al., 2008; Goel et al., 2009; Jackson et al., 2016; McMahon et al., 2018); however, the ability to sustain attention is regarded as one of the cognitive functions most sensitive to fatigue, with deficits manifesting after a single night of extended wakefulness (Wilson et al., 2007; Franzen et al., 2008; Slama et al., 2018; Mantua et al., 2021) and progressively deteriorating with increasing time awake (Doran et al., 2001; Van Dongen et al., 2003b). In real-life situations, it has also been observed that scores on a cognitive function task decrease after an on-call shift (Halbach et al., 2003).

Slower reaction times are a consistent effect of increasing time awake, an effect that can be observed in a widespread number of measures (i.e., Serial Reaction Time tasks, Pigeau et al., 1995; Axelsson et al., 2008; Stimulus Reaction tasks, Philip et al., 2004; Romeijn et al., 2012). However, sleep deprivation paradigms often study sustained attentional states by using the PVT, a simple, yet attentionally-demanding task that requires participants to continuously detect and respond to randomly occurring stimuli. The widespread use of the PVT has yielded useful insights into the impact of extended wakefulness on sustained attention, which is characterized by an increase of attentional lapses, as well as an overall slowing of reaction time (Lim and Dinges, 2008). Furthermore, PVT is often used to study vigilant attention and alertness. These effects are also evident when using other types of attentionally-demanding tasks (i.e., Simple Reaction Time Task; Detection Tasks; Task Switching Tasks, Stroop Test), a trend that demonstrates the robust impact of sleep deprivation on attention (Dodds et al., 2011; Philip et al., 2012; Deliens et al., 2018; McMahon et al., 2018). Further, other forms of attention have also manifested fatigue-related effects; for instance, McMahon et al. (2018) found that complex attention, as measured by the Identification Task, was impaired after 27 h of being awake. Similarly, Chua et al. (2017) tested the effects of fatigue on divided attention and found that participants experienced substantial impairments in dividing their attention between tasks of varying complexity after 40 h of continuous wakefulness. In real-world studies, it has been found that medical residents exhibit an increase in reaction time on vigilance tests following night duty as well as a decrease in alertness (Orton and Gruzelier, 1989; Howard et al., 2003; Bartel et al., 2004). Additionally, decreased vigilance and a decrease in response speed has also been observed in pilots (Neri et al., 2002; Bourgeois-Bougrine et al., 2003). Furthermore, Lockley et al. (2004) found that medical interns experience an increase in attentional failures after working a night shift. These attentional failures doubled with interns who were forced to work more hours (approximately 84.9 h per week) compared to those who got to work significantly less (approximately 65.4 h per week).

Linked to attention, response inhibition has also demonstrated susceptibility to fatigued states (Chuah et al., 2006; Drummond et al., 2006; Deliens et al., 2018). To test this, Drummond et al. (2006) asked participants to perform a Go/No-Go task throughout two nights of sleep deprivation. Go/No-Go tasks require participants to respond to a set of stimuli as quickly as possible and withhold responses when presented with a predetermined target. Their results demonstrated that hit accuracy decreased and response time increased after 55 h of continuous wakefulness, suggesting that fatigue interferes with the ability to withhold incorrect responses. Similarly, Slama et al. (2018) found decreases in response inhibition and increases in response times on the Stop Signal task after a night of sleep deprivation, whereas participants in the rested condition did not exhibit such effects.

Beyond attention, research has shown that sleep deprivation also affects cognitive control, flexible thought processing (Bratzke et al., 2009; Deliens et al., 2018; Slama et al., 2018), working memory (Durmer and Dinges, 2005; Dodds et al., 2011; Deliens et al., 2018), and arithmetic ability (Drummond and Brown, 2001; Boardman et al., 2018). Flexible thought processing (i.e., developing novel, divergent inferences) is an essential part of solving complex problems and unpredictable events that cannot be solved using logical, deductive reasoning. Interestingly, when participants are given tasks that require them to engage in flexible thinking, sleep deprivation manifests more adverse effects than when they perform logical, rule-based tasks (Harrison and Horne, 2000). For example, Couyoumdjian et al. (2010) compared task-switching performance between sleep-deprived and well-rested participants. Task-switching paradigms consist of performing two different tasks in rapid, random succession, often resulting in high switch-costs (i.e., longer reaction times). Their results demonstrated that participants in the sleep deprivation group had significantly more accuracy errors, higher switch costs, and slower reaction times compared to the rested group. Similarly, Deliens et al. (2018) found higher switching costs using a within-subjects design, such that participants took part in both well-rested and sleep deprivation conditions.

Fatigue-related deficits are also present when tasks draw on memory; therefore, many sleep deprivation paradigms are focused on different types of memory. For instance, short-term memory is often studied by using digit span tests (Pigeau et al., 1995) and repeated acquisition tests (Lieberman et al., 2002); these tests yield results that indicate a decline in short-term memory. Concerning working memory deficits, common measurement tasks include the N-Back task and arithmetic tasks. Fatigued participants consistently exhibit lower accuracy and increased reaction times on the N-Back task (Dodds et al., 2011; Deliens et al., 2018; McMahon et al., 2018; Slama et al., 2018). Furthermore, working memory is also studied by using spatial span and pattern recognition tasks (Dodds et al., 2011); these tests show declines in working memory following sleep deprivation. Others have demonstrated that these deficits can also be seen in arithmetic tasks, which heavily rely on working memory capacity; Boardman et al. (2018) demonstrated this by asking participants to solve equations with varying levels of difficulty throughout 66 h of continuous wakefulness. Their results showed that fatigued participants had poorer accuracy overall, which worsened with increasing task difficulty when compared to being in a rested state. Applied research has found that medical residents on the night shift exhibit a decline in visual memory capacity as measured by the Delayed Recognition Span Test (Rollinson et al., 2003). However, no impact on short-term attention (Rollinson et al., 2003) or the ability to learn medically relevant information (Browne et al., 1994) was observed. Furthermore, it has been observed in the workplace that fatigued employees tend to be less productive and don’t perform as well (Rosekind et al., 2010).

Interestingly, changes in cognition don’t always point back to fatigue. Changes in cognition could be due to other confounding factors. For instance, increases in attentional lapses are often associated with fatigue, but it could also be due to factors such as being in an environment with a higher cognitive load (Buckley et al., 2016), being bored (Cheyne et al., 2006), and mind wandering (Chernyshev et al., 2015). Additionally, a decrease in reaction time can be associated with fatigue, but it can also be associated with age (Hultsch et al., 2002), distraction due to background noise (Trimmel and Poelzl, 2006), and alcohol impairment (Hernandez et al., 2007).

### Fatigue and behavior

Fatigue has been associated with various impacts on behavior (see Table 1). Most notably, it leads to variations in mood states. Past work has suggested that it affects mood states more intensely than it does motor and cognitive performance (i.e., higher scores on mood scales when fatigued; Pilcher and Huffcutt, 1996). Sleep deprivation often leads to an increase in negative emotions (Chuah et al., 2006; Tempesta et al., 2010), anger, and confusion (Paterson et al., 2011; Schwarz et al., 2019), as well a decrease in positive affect (Zohar et al., 2005; Franzen et al., 2008; Schwarz et al., 2019). Pigeau et al. (1995), found that after 48 h of sleep deprivation, individuals experienced a greater decrease in mood states compared to after only 24 h, signifying that mood states gradually worsen the longer an individual is awake. Similarly, Franzen et al. (2008) found correlations between increases in subjective sleepiness and decreases in mood states, suggesting that participants experience worsening moods as their feelings of sleepiness increase.

**TABLE 1:** Summary of the types of measurements for different types of effects of fatigue on behavior.

Fatigue related variations in performance	Domain	Sub-domain	Effect	Measurements	Representative citation
Effects on behavior	Mood/emotions		Decline in mood and increase in negative emotions (depression, anxiety, paranoia, etc.)	Mood scale	Pigeau et al., 1995; Chuah et al., 2006; Paterson et al., 2011
				Profile of mood States (POMS)	Newhouse et al., 1989; Orton and Gruzelier, 1989; Lieberman et al., 2002; Howard et al., 2003; Zohar et al., 2005; Wilson et al., 2007; Previc et al., 2009; Minkel et al., 2012; Michael et al., 2013; McIntire et al., 2014, 2017; Schwarz et al., 2019; Abe et al., 2020
				International affective picture system	Tempesta et al., 2010; Pilcher et al., 2015
				Likert scale	Chuah et al., 2006; Deliens et al., 2018
				Mood States questionnaire	Bartle et al., 1988
				Sleep deprivation survey	Ranasinghe et al., 2018
				Positive and negative affect schedule (PANAS)	Zohar et al., 2005; Franzen et al., 2008; Gerhardsson et al., 2019; Schwarz et al., 2019
				Personality assessment inventory	Kahn-Greene et al., 2007
			Decrease in positive thinking	Bar-On emotional quotient inventory	Killgore et al., 2008
			Decrease in motivation	Mood scale	Pigeau et al., 1995
				PANAS	Franzen et al., 2008
				Likert scale	Chuah et al., 2006
	Emotional perception/ responding to stressful situations	Responsivity	Increased responsivity	International affective picture system	Tempesta et al., 2010; Pilcher et al., 2015
		Pupillary response to emotional stimuli	Increased pupil dilation	Pupilometer	Franzen et al., 2009
	Interpersonal skills	Emotional intelligence	Decrease in emotional intelligence scores	Bar On emotional quotient inventory	Killgore et al., 2008
		Social skills	Decrease in social skills	Visual perspective taking tasks	Deliens et al., 2018
		Personality	Change in personality	Personality assessment inventory	Kahn-Greene et al., 2007
	Impulsivity		Increase in impulsivity	Risk taking assessment	Choshen-Hillel et al., 2021
				Balloon analog risk task	Killgore et al., 2011
				Evaluation of risks scale	Killgore et al., 2011
				Iowa gambling task	Killgore et al., 2006b
	Behavioral performance	Simulations	Decreased performance on simulations	Number of flight simulation errors	Previc et al., 2009
				Simulated laparoscopic surgery	Howard et al., 2003
				Driving simulatory test	Rowland et al., 2005; Jackson et al., 2016
		Task performance	Decline in task performance	Target identification	McKinley et al., 2011
				Rifle marksmanship	Lieberman et al., 2002
				Truck driver reports	Garbarino et al., 2016
		Motor coordination and speed	Decline in coordination and speed	The grooved pegboard	Halbach et al., 2003; Killgore and Kamimori, 2020a

In sleep deprivation paradigms related to mood, the Profile of Mood States (POMS) is often used. Results on this measure frequently demonstrate that as fatigue increases, mood tends to worsen (Lieberman et al., 2002; Wilson et al., 2007; Michael et al., 2013; McIntire et al., 2014, 2017; Abe et al., 2020), a trend that has also been found when using other measurements such as the Mood Scale (Chuah et al., 2006), a Seven-Point Likert scale (Deliens et al., 2018), and the Positive and Negative Affect Schedule (PANAS; Gerhardsson et al., 2019). More specifically, declines in mood resulting from fatigue lead to increases in depression, confusion (Lieberman et al., 2002), irritability, and anxiety (Durmer and Dinges, 2005). These results have been found in laboratory studies as well as applied studies. For instance, prior work has observed fluctuations in the mood states of medical professionals following night and extended shifts. For example, Orton and Gruzelier (1989) examined variations in mood states in young doctors after extended shifts (i.e., lasting up to 31 h). Interns and residents tend to experience a decrease in mood (i.e., increased feelings of anger and confusion; decreased feelings of motivation and happiness) following both night and extended shifts (Bartle et al., 1988; Orton and Gruzelier, 1989; Browne et al., 1994; Howard et al., 2003). Additionally, sleep deprivation observed in college students leads to an increase in irritability, lack of motivation, and an increase in mood swings (Ranasinghe et al., 2018).

Evidence has also shown that fatigue influences emotional perception. To illustrate, Tempesta et al. (2010) presented subjects with pleasant, neutral, and unpleasant stimuli and asked them to rate their emotional quality. Next, participants underwent a night of normal sleep or one night of sleep deprivation and rated a matched set of stimuli. Results showed that subjects rated neutral stimuli as significantly more negative following sleep deprivation. These results were replicated by Pilcher et al. (2015), suggesting an emotional processing bias toward negativity due to fatigue. Fatigue has also been linked with increases in clinical symptoms associated with psychopathology (Kahn-Greene et al., 2007). For instance, Kahn-Greene et al. (2007) found that sleep-deprived subjects reported significant increases on clinical scales measuring depression, anxiety, and paranoia, relative to their baseline responses, suggesting a profound impact of sleep deprivation on emotional functioning.

Past work has shown that fatigue also influences emotional self-regulation and coping mechanisms. For instance, Killgore et al. (2008) found that two nights of sleep deprivation leads to lower emotional intelligence scores (i.e., reduced self-regard, assertiveness, sense of independence, and self-actualization), decrease in interpersonal functioning (i.e., reduced empathy toward others), increased impulsivity, decreased behavioral coping, and reduced positive thinking. Sleep deprivation has also been found to affect responses to stressful conditions. Minkel et al. (2012) studied this by manipulating the level of stress intensity (i.e., low vs. high stress) on a task after a night of sleep deprivation. Results showed that fatigued participants reported greater subjective stress in the low-stress condition relative to well-rested participants, suggesting that sleep deprivation alters the threshold at which a person perceives a certain event as stressful.

Being in a fatigued state also influences judgment and risk-taking processes, as fatigued individuals tend to view short-term rewards as more valuable than long-term rewards, which relates to difficulties with delaying gratification. Killgore et al. (2006b) exemplified this in a study that examined the effects of 49 h of sleep deprivation on an emotionally guided task (i.e., Iowa Gambling Task) that required participants to delay gratification to ensure long-term success. Well-rested participants preferred to avoid high-risk choices in order to ensure consistent rewards; however, the same subjects preferred to make riskier selections after two nights of sleep deprivation. Killgore et al. (2011) found a similar trend when using the Balloon Analog Risk-Taking task, such that participants engaged in increased risk-taking behaviors after 75 h of sleep deprivation. In both the Balloon Analog Risk Task and Iowa Gambling Task, fatigued participants were more likely to make risky decisions. The behavioral effects of fatigue have also been identified in real-life settings, such that the judgment of an individual is impacted. For instance, Choshen-Hillel et al. (2021) found that the judgment of medical residents was impacted. They found an increase in impulsivity and slower processing time when making decisions after a 26-h shift when compared to baseline levels (i.e., when well-rested). Consequently, fatigue-related variations in behavior can lead to effects on everyday decision-making, as these processes not only rely on cognitive capacity, but also emotional factors (Killgore, 2010).

Fatigue also impacts task performance. Errors on simulation tasks are often used to gauge performance deficits. For instance, Previc et al. (2009) found that increasing wakefulness degrades the ability to accurately perform tasks in a flight simulator. Ten military pilots flew a series of flight simulations over a 34 h period of sleep deprivation during which flying errors were evaluated. Results demonstrated increasing flight errors peaking at approximately 24 h of wakefulness, which were in line with subjective reports of sleepiness. Similar effects have been found in other simulated settings. Jackson et al. (2016) asked professional drivers to complete a series of simulated driving tasks after a night of rest and after a night of sleep deprivation. Their results demonstrated that participants had increased variations in speed, lane positioning, and attentional lapses, as well as decreases in braking reaction time. These findings are also evident in real-life settings. For instance, Lockley et al. (2004) found that medical interns experienced an increase in attentional failures during extended work shifts and night hours (approximately 84.9 h per week) compared to a work schedule with fewer hours (approximately 65.4 h per week). Additionally, medical residents working extended or night shifts experience a decline in performance, dexterity, and surgical skills compared to residents who received a couple of extra hours of sleep per night (Veasey et al., 2002; Howard et al., 2003). Furthermore, it has been observed that in commercial truck drivers, sleep deprivation leads to a decrease in ability to perform as seen by an increase in accidents and an increase in near-accidents (Garbarino et al., 2016). An increase in accidents due to fatigue can also be seen in aviation (Caldwell et al., 2009). However, changes in performance are not unique to fatigue. Changes in performance could also be due to variables such as alcohol impairment (Shiferaw et al., 2014) or distracting environments (Nabatilan et al., 2012).

### Fatigue and physiology

Sleep deprivation is known to induce a wide range of physiological effects (see Table 2). For instance, it has been associated with variations in cardiovascular functions, metabolism, ocular activity, thermoregulation, and brain activity. Sleep deprivation leads to alterations in the autonomic nervous system (ANS; Burgess et al., 1997; Ogawa et al., 2003; Tobaldini et al., 2013; Glos et al., 2014); more specifically, it leads to an increase in sympathetic activity (Holmes et al., 2002; Zhong et al., 2005) and a decrease in parasympathetic activity (Zhong et al., 2005; Tobaldini et al., 2013). In particular, fatigue-related variations have been linked to the cardiac autonomic nervous system. For instance, sleep deprivation leads to decreases in heart rate variability (HRV; Zhong et al., 2005; Glos et al., 2014), which is considered a useful, non-invasive measure of cardiac ANS state. Interestingly, Chua et al. (2012) found that changes in HRV are correlated with increases in PVT lapses (i.e., decreases in attention) suggesting that changes in HRV states could be used to predict an individual’s vigilance level. Furthermore, fatigued individuals exhibit increased blood pressure (Kato et al., 2000; Ogawa et al., 2003; Patrick et al., 2017), decreased muscle sympathetic nerve activity (MSNA; Kato et al., 2000; Ogawa et al., 2003), and a decreased heart rate (HR; Burgess et al., 1997; Holmes et al., 2002; Zhong et al., 2005; Vaara et al., 2009). Additionally, increased blood pressure, resulting from fatigue, has been observed in real-world settings with commercial long-haul truck drivers (Stoohs et al., 1995). Substantial evidence has shown that sleep deprivation has a perturbing effect on cardiovascular balance; however, there have been reports of physiological processes that may serve as protection mechanisms against the stress caused by sleep deprivation. Namely, Vaara et al. (2009) found that the disruption of cardiac sympathetic balance imposed by sleep deprivation led to decreases in HR and increases in vagal nerve activity. These physiological responses have been associated with autonomic regulation processes in response to sleep deprivation.

**TABLE 2:** Summary of the types of measurements for different types of effects of fatigue on physiology.

Fatigue related variations in performance	Domain	Sub-domain	Effect	Measurements	Representative citation
Effects on physiology	Autonomic nervous system	Sympathetic activity	Increase in sympathetic activity	Determined by the heart rate and pre ejection period (using ECG)	Holmes et al., 2002; Zhong et al., 2005; Viola et al., 2008
		Parasympathetic activity	Decrease in parasympathetic activity	Determined by the heart rate (using ECG)	Zhong et al., 2005; Viola et al., 2008; Tobaldini et al., 2013
		Muscle sympathetic nerve activity (MSNA)	Decrease in MSNA	Epidural catheter (to measure postganglionic sympathetic activity)	Kato et al., 2000
				Tungsten microelectrode (inserted in peroneal nerve)	Ogawa et al., 2003
	Respiratory	Breathing rate	Breathing becomes more shallow	Respiration belt	Ogilvie et al., 1989; Holmes et al., 2002; Barry et al., 2005
				Abdominal amplitude	Ogilvie and Wilkinson, 1984
				Spirometry	Patrick et al., 2017
	Cardiovascular	Heart rate variability (HRV)- Low frequency (LF) aspect	Increase in LF aspect of HRV	ECG	Zhong et al., 2005; Tobaldini et al., 2013
				Wrist monitor	Vaara et al., 2009
			Decrease in LF aspect of HRV	ECG	Glos et al., 2014
		Heart rate variability (HRV)- High frequency (HF) aspect	No change in HF aspect of HRV	ECG	Glos et al., 2014
			Decrease in HF aspect of HRV	ECG	Zhong et al., 2005; Tobaldini et al., 2013
			Increase in HF aspect of HRV	Wrist monitor	Vaara et al., 2009
		Heart rate	Decrease in heart rate	Wrist monitor	Vaara et al., 2009
				ECG	Burgess et al., 1997; Holmes et al., 2002
			Increase in heart rate	ECG	Chua et al., 2012
			No changes in heart rate	ECG	Kato et al., 2000
		Pre-ejection period (PEP)	Increase in pre-ejection period	ECG	Burgess et al., 1997; Holmes et al., 2002
		Blood pressure	No changes in blood pressure variability (BPV)	Sphygnomanometer	Kato et al., 2000; Tobaldini et al., 2013
				Blood pressure autonomic monitor	Vaara et al., 2009
			Increase in blood pressure	Non-invasive device	Patrick et al., 2017
				Non-invasive tonometric device	Ogawa et al., 2003; Zhong et al., 2005
				Sphygmomanometer	Stoohs et al., 1995
	Thermoregulation	Body temperature	Increase in core body temperature fluctuations	Rectal thermometer	Holmes et al., 2002
				Ingestible vitasense temperature transmitter	Chua et al., 2012
			Increase in core body temperature	Vitasense temperature transmitter	Słomko et al., 2018
			Decrease in body temperature	Ear-in thermometer	Vaara et al., 2009
	Neurological	Variations in cerebral metabolism	Decrease in anterior cingulate cortex (ACC) metabolic activity	Electroencephalography (EEG)	Hsieh et al., 2007
			Decrease in global and regional (prefrontal, parietal, cortices, and thalamus) cerebral glucose metabolic rate (CMR)	Positron emission tomography (PET)	Wu et al., 1991; Thomas et al., 2000
			Increase in visual cortex cerebral glucose metabolic rate	PET	Wu et al., 1991
			Decrease in glucose metabolic measurements in the thalamus, basal ganglia, white matter, and cerebellum	PET	Wu et al., 1991
			Increase in cerebral blood oxygen levels	Functional magnetic resonance imaging (fMRI)	Drummond et al., 2000, 2005
		Brain waves	Decrease in alpha activity	EEG	Ogilvie and Wilkinson, 1984; Wilson et al., 2007; Previc et al., 2009
			Increase in alpha activity	EEG	Gorgoni et al., 2014
			Increase in beta activity	EEG	Gorgoni et al., 2014
			Increase in theta activity	EEG	Finelli et al., 2000; Neri et al., 2002; Previc et al., 2009; Gorgoni et al., 2014
			Increase in delta activity	EEG	Ogilvie and Wilkinson, 1984; Wilson et al., 2007; Previc et al., 2009; Gorgoni et al., 2014
			Increased degree of band connectivity	EEG	Kar et al., 2011
		Brain structure	Increase density of gray matter (central, prefrontal, ACC)	High resolution T1 structural scans (MRI)	Sun et al., 2020; Killgore et al., 2020b
			Decrease density of gray matter (thalamus and temporal regions)	High resolution T1 structural scans (MRI)	Liu et al., 2014; Sun et al., 2020
			Decrease cortical thickness (temporal and parietal)	High resolution T1 structural scan (MRI)	Elvsåshagen et al., 2017; Sun et al., 2020
		Brain activity	Decrease in ventral and prefrontal activation	fMRI	Chuah et al., 2006
			Decreased activity in parietal lobes and premotor areas	fMRI	Drummond et al., 1999
			Decreased activity in the prefrontal cortex	fMRI	Drummond et al., 1999
			Decreased global brain activation	fMRI	Mu et al., 2005
			Decreased white matter in areas susceptible to sleep deprivation	fMRI	Rocklage et al., 2009
			Increased white matter in areas not susceptible to sleep deprivation	fMRI	Rocklage et al., 2009
	Metabolic	Hormone levels	No difference in hormone levels	Blood sample	Tobaldini et al., 2013
				Plasma sample	Ogawa et al., 2003
		Biomarkers	Increase in biomarkers	Blood sample	Glucose, creatine, serum urea: Kar et al., 2011 High sensitivity c-reactive protein: Choshen-Hillel et al., 2021
				Saliva sample	Melatonin: Michael et al., 2013; Abe et al., 2020
		Cytokine levels	No difference in tissue inflammatory cytokine levels	Blood sample	Tobaldini et al., 2013
	Ocular	Pupil measurements	Increase in pupil diameter	Pupil sleepiness test	Franzen et al., 2008
				Pupilometer	Franzen et al., 2009
			Decrease in pupil diameter	Eye-tracking system (EMR-9)	Abe et al., 2011, Abe et al., 2020
			No significant change in pupil diameter	FIT-2500 fatigue analyzer	Goldich et al., 2010
			Increase in pupil latency	Automated oculomotor test	Rowland et al., 2005
			Increase in pupil dilation	Pupil sleepiness test	Franzen et al., 2008
				Pupilometer	Franzen et al., 2009
			Increase in pupil area	EyeLink II system	Wilson et al., 2007
		Eye movement	No change in instrument scanning	Eye-Trac 6000	Previc et al., 2009
			Increase in slow eye movement	EOG	Neri et al., 2002
		Eyelid closure	Increase in blink duration	EOG	Morris and Miller, 1996; Abe et al., 2020
				Infrared reflectance oculography (Optalert)	Ftouni et al., 2013
			Increase in percentage of eyelid closure over time (PERCLOS)	Eye tracking system (EMR-9)	Abe et al., 2011, 2020
				EyeCom eye tracker (EC6)	McKinley et al., 2011
				ISCAN eye tracker	Chua et al., 2012
				Copilot eye tracker	Jackson et al., 2016
				EOG	Chua et al., 2012
				Video camera	Dinges et al., 1997
			Increased percentage of time with eyes closed	Infrared reflectance oculography (Optalert)	Ftouni et al., 2013; Wilkinson et al., 2013
			Decreased blink frequency	EOG	Abe et al., 2011; Chua et al., 2012
			Increase in eye blink rate	Spontaneous eye blink rate recording (sEBR)	Slama et al., 2018
				EOG	Morris and Miller, 1996
		Saccades and Microsaccades	Decrease in saccade velocity	EOG	Morris and Miller, 1996
				Fitness impairment tester	Goldich et al., 2010
				Electronystagm (ENG)	Fransson et al., 2008
			Increase in number of saccades	250-Hz eye tracker	Stone et al., 2019
			Decrease in number of microsaccades	EMR-9 eye tracker	Abe et al., 2020
				EOG	Abe et al., 2020

Sleep deprivation is known to affect different functional aspects of brain activity. Typical sleep deprivation studies use neuroimaging devices such as Functional Magnetic Resonance Imaging (fMRI), Positron Emission Tomography (PET), and Magnetoencephalography (MEG) to detect changes in brain networks. For instance, sleep loss has been linked to variations in cerebral metabolism. Hsieh et al. (2007) identified fatigue-related decreases in anterior cingulate cortex (ACC) metabolic activity, which is associated with decreased executive functioning. Wu et al. (1991) found significant decreases in global cerebral glucose metabolic rate (CMR), a marker for neuronal activity, after 32 h of sleep deprivation. This decrease was coupled with performance deficits on a Continuous Performance Test, which measures visual vigilance. Another study also found decreased global CMR and decreases in regional CMR, specifically in prefrontal and parietal cortices, as well as in the thalamus (Thomas et al., 2000), which were concurrent with decreases in alertness and cognitive performance. Similarly, Chuah et al. (2006) found decreased ventral and prefrontal activation in sleep-deprived participants when attempting to successfully perform a Go/No-Go task. There is also evidence suggesting that the prefrontal cortex (PFC), a region devoted to executive functioning, is particularly susceptible to fatigue. (Drummond et al., 1999, 2000; Harrison and Horne, 2000; Thomas et al., 2000; Goel et al., 2009; Deliens et al., 2018). For instance, Drummond et al. (2000); Drummond and Brown (2001), and Drummond et al. (2005) reported finding greater cerebral blood oxygen levels in the bilateral prefrontal and parietal cortex following a period of total sleep deprivation during verbal learning and divided attention tasks, suggesting that the brain recruits multiple brain regions as a compensatory response to performing tasks that require executive functioning while sleep deprived. Similar suggestions have been reported previously by Harrison and Horne (1998), such that sleep-deprived subjects were significantly impaired when performing verbal and unfamiliar tasks. Sleep deprivation is also associated with variations in both localized (Ogilvie and Wilkinson, 1984; Wilson et al., 2007; Previc et al., 2009) and global (Caldwell et al., 2004a; Gorgoni et al., 2014) electroencephalogram (EEG) activity; namely decreases in alpha waves and increases in delta and theta band power. Furthermore, Gorgoni et al. (2019) has identified specific increases in beta activity in the fronto-central midline area of the brain while Finelli et al. (2000) identified increases in theta activity in the frontal region of the brain.

Research has also revealed the effects of sleep deprivation on structural aspects of the brain. Killgore et al. (2020b) identified fatigue-related effects in ventral, prefrontal, and anterior cingulate areas of the brain; specifically, they found that increasing lapses on the PVT correlated with greater density of gray matter in these areas of the brain. Following a night of sleep deprivation, an increase in gray matter density was observed in the PFC (Sun et al., 2020); whereas, a decrease in gray matter density was observed in the thalamus (Liu et al., 2014) and temporal regions of the brain (Sun et al., 2020). Changes in cortical thickness have also been observed such that a night of sleep deprivation leads to a decrease in cortical thickness in the temporal (Sun et al., 2020) and parietal (Elvsåshagen et al., 2017) regions of the brain.

Fatigued states can also be assessed through a wide array of ocular behavior. Prior research has studied fatigue-related effects that range from variations in pupil size to percentage of eyelid closure (PERCLOS). For instance, Franzen et al. (2009) examined variations in pupil diameter when viewing emotional stimuli following a night of sleep deprivation. The pupil is known to dilate in response to emotional information (Franzen et al., 2009). However, compared to fully rested participants, fatigued individuals had larger degree of pupil dilation when viewing emotional stimuli and exhibited an anticipatory pupil reaction when expecting to view negative stimuli. These results suggest that sleep deprivation may lead to heightened responses to negative stimuli. Other common ocular measurements include blink duration and activity, saccades, eye and eyelid movement, and the duration of eyelid closure. Previous research has identified that ocular measures are fairly accurate at predicting performance deficits due to fatigue (Ftouni et al., 2013); more specifically, PERCLOS was found to be a valid measure of drowsiness (Abe et al., 2011; Jackson et al., 2016). Jackson et al. (2016) studied the effects of fatigue on PERCLOS during a simulated driving task. After a period of sleep deprivation, participants exhibited a greater amount of eyelid closure (eyes were closed for longer periods of time) compared to fully rested participants. Furthermore, PERCLOS is correlated with a decrease in performance and an increase in PVT lapses and fatigue (Dinges et al., 1997; McKinley et al., 2011; Chua et al., 2012; Jackson et al., 2016). Other changes in eye movement are also associated with fatigue. McKinley et al. (2011) found that fatigued individuals exhibit slower eye movements with less irregularity. Furthermore, Neri et al. (2002) conducted a real-life experiment on pilots and also observed an increase in slow eye movement. Stone et al. (2019) examined 14 oculomotor measures and found that a majority were significantly impaired by fatigue and modulated by circadian rhythms. More specifically, the number of blinks and saccadic rate increased; whereas, eye acceleration, steady-state gain, proportion smooth (i.e., portion of time that eye-tracking was smooth), and saccadic velocity decreased. Similar findings had been put forth by Caldwell et al. (2004a), such that saccadic velocity decreased as the level of sleep loss in their subjects increased. These consistent variations suggest that using a comprehensive number of oculometric measurements could be used as a biomarker of fatigue (Stone et al., 2019). However, it is important to note that oculometric measurements are indicative of more than just fatigue. For instance, blink frequency could indicate an increase of fatigue, but it could also be due to a decrease in cognitive load (Svensson and Wilson, 2002) or a decrease in complexity of the environment (Faure et al., 2016). Therefore, oculometric measurements should be used in conjunction with other measurements in order to more accurately detect fatigue.

#### Circadian rhythms

An important element of studying the effects of sleep deprivation is to identify effects that result from a disruption of circadian rhythms. It is possible that the effects seen during a night of sleep deprivation are the result of a disruption of circadian patterns. Circadian disruptions have been used to account for many of the physiological effects seen following sleep deprivation. For instance, disruption of circadian rhythms due to sleep deprivation has been shown in autonomic nervous system processes, having the strongest influence on the cardiac system, resulting in a decrease in heart rate and an increase in heart rate variability (Viola et al., 2008) as well as an increase in blood pressure variability during the early hours of the day, following a night of sleep deprivation (Zhong et al., 2005; Glos et al., 2014). Burgess et al. (1997) found that the parasympathetic system is affected by circadian rhythms, as evidenced by changes in respiratory sinus arrhythmia (RSA) measurements when controlling for the influence of sleep. Other physiological aspects have been found to have a circadian influence as well. For instance, Michael et al. (2013) found that some salivary biomarkers (i.e., the fatigue biomarker index) exhibited similar patterns of change, regardless of whether participants were sleep deprived or rested, suggesting a circadian influence. Sinusoidal circadian patterns have also been observed in ocular measures such as pursuit behavior, saccade behavior, latency, proportion smooth, smooth pursuit accuracy, and PERCLOS (Fransson et al., 2008; Ftouni et al., 2013; Stone et al., 2019).

### Fatigue and subjective sleepiness

A well-replicated effect of sleep deprivation is that individuals report progressively greater levels of subjective sleepiness as time awake increases (Rowland et al., 2005; Wesensten et al., 2005; Chuah et al., 2006; Wilson et al., 2007; Goldich et al., 2010; Abe et al., 2011; Gorgoni et al., 2014; Jackson et al., 2016; Deliens et al., 2018; Slama et al., 2018; Gerhardsson et al., 2019). Subjective sleepiness assesses how tired an individual feels at a given point in time. Many sleep deprivation paradigms utilize scales such as the Karolinska Sleepiness Scale (KSS), Stanford Sleepiness Scale (SSS), and the Visual Analog Scale (VAS) to determine subjective sleepiness, as these scales accurately portray increases in subjective sleepiness and feelings of fatigue following sleep deprivation (Philip et al., 2012; Lo et al., 2016; Paech et al., 2016). However, other, similar measures have also been identified in the literature (see Table 3 for a summary). An increase in subjective sleepiness is also observed in real-life conditions. For instance, sleep-deprived medical residents experienced increased feelings of subjective feelings of sleepiness and fatigue following a night shift (Bartle et al., 1988; Browne et al., 1994).

**TABLE 3:** Summary of the types of measurements for different types of effects of fatigue on subjective measures.

Fatigue related variations in performance	Domain	Effect	Measurements	Representative citation
Effects on subjective measures	Subjective sleepiness	Increase in subjective sleepiness	Karolinska sleepiness scale (KSS)	Neri et al., 2002; Van Dongen et al., 2003b,^2004^; Philip et al., 2004, 2012; Drummond et al., 2005; Chuah et al., 2006; Bratzke et al., 2009; Anderson and Dickinson, 2010; Abe et al., 2011, 2020; Chua et al., 2012; Ftouni et al., 2013; Glos et al., 2014; Gorgoni et al., 2014; Jackson et al., 2016; Lo et al., 2016; Boardman et al., 2018; Deliens et al., 2018; Gerhardsson et al., 2019; McMahon et al., 2018; Schwarz et al., 2019; Slama et al., 2018; Sun et al., 2020; Cheng et al., 2021
			Stanford sleepiness scale (SSS)	Ogilvie et al., 1989; Penetar et al., 1993; Pigeau et al., 1995; Morris and Miller, 1996; Lieberman et al., 2002; Van Dongen et al., 2003b; Bartel et al., 2004; Drummond et al., 2005; Rowland et al., 2005; Wesensten et al., 2005; Kohler et al., 2006; Hsieh et al., 2007; Goldich et al., 2010; Mollicone et al., 2010; Killgore and Kamimori, 2020a; Cheng et al., 2021
			Visual analog scale (VAS)	Penetar et al., 1993; Neri et al., 2002; Philip et al., 2004; Van Dongen et al., 2004; Kohler et al., 2006; Wilson et al., 2007; Fransson et al., 2008; Franzen et al., 2008; Previc et al., 2009; Tempesta et al., 2010; Abe et al., 2011; Dodds et al., 2011; Chua et al., 2012; Minkel et al., 2012; McIntire et al., 2014, 2017; Slama et al., 2018; Cheng et al., 2021
			Fatigue questionnaire	Bourgeois-Bougrine et al., 2003
			9- Point Likert scale assessing subjective sleepiness	Paech et al., 2016
			Pittsburg sleep quality index	Couyoumdjian et al., 2010; Dodds et al., 2011; Choshen-Hillel et al., 2021
			Connor-Davidson resilience scale	Mantua et al., 2021
			Epworth sleepiness scale	Garbarino et al., 2016
			Linear analog scales	Boyle et al., 2012
			Sleep habits questionnaire	Stoohs et al., 1995
			Sleep deprivation survey	Ranasinghe et al., 2018
	Subjective mood	Decline in subjective mood	PANAS	Zohar et al., 2005; Franzen et al., 2008; Gerhardsson et al., 2019; Schwarz et al., 2019
			Mood questionnaire	Bartle et al., 1988; McIntire et al., 2017
			Side effects questionnaire	McIntire et al., 2014, 2017; Killgore and Kamimori, 2020a
	Subjective performance	Decline in subjective performance	Evaluation of risks sale	Killgore et al., 2011
			Likert scale assessing subjective performance	Howard et al., 2003; Paech et al., 2016; Boardman et al., 2018

Interestingly, each individual rates their own subjective sleepiness differently suggesting differences in levels of resilience to sleep deprivation (Philip et al., 2004; Van Dongen et al., 2004; Killgore et al., 2011; Short and Banks, 2014). For example, these variations are present between age groups, such that older and younger adults differ in how they perceive their own levels of sleepiness. For instance, Philip et al. (2004) found that younger adults reported feeling less tired than they objectively were, as demonstrated by their reaction time performance. Namely, the inaccuracy of young adults self-perception of sleepiness was evident by their increased performance decrements (i.e., longer reaction time) compared to older adults, who more accurately estimated how their feelings of sleepiness would impact their objective performance. Since individuals possess different levels of resistance to fatigue, having an understanding of how performance is impacted varies from person to person, therefore making subjective sleepiness an unreliable measure of objective fatigue-related performance deficits by itself. For instance, Mantua et al. (2021) found that subjective sleep didn’t predict performance on PVT (i.e., reaction time and lapses). Although subjective sleepiness cannot be solely used to determine an individual’s level of sleepiness, it can be used in combination with other measures to detect and predict fatigue-related deficits. For instance, prior research has used subjective measures in combination with physiological effects to predict fatigue. Wilson et al. (2007) suggested that both physiological and subjective data parallel fatigue-related changes in performance. They found that a decline in performance on PVT (i.e., increased reaction time) was associated with an increase of delta and theta activity on the EEG (lower frequency waves associated with decreased alertness) as well as increased subjective feelings of fatigue and sleepiness. Furthermore, other research has found correlations between subjective sleepiness and oculomotor measures, suggesting that subjective sleepiness, in combination with some oculomotor measures, may be used to detect when an individual is approaching a state of fatigue (Rowland et al., 2005; Goldich et al., 2010).

## Mitigating fatigue

With the growing demands of society, people increasingly seek out ways to mitigate the effects of fatigue. The most common and accessible method is through the use of caffeine, which is often used in research settings since it is readily available to the public. Other drugs, such as modafinil, d-amphetamine, Donepezil, and AMPAKINE compounds (CX717), have also been studied for their mitigation properties. Each drug has a different elimination rate which causes differences in the time it takes post-administration to have its effect and in the duration of benefits (Wesensten et al., 2005). Furthermore, other methods such as tDCS and transcutaneous vagal nerve stimulation (tVNS; McIntire et al., 2014) have been studied as a way to stimulate the brain non-invasively. Table 4 summarizes the different types of mitigation strategies.

**TABLE 4:** Summary of different types of fatigue mitigation.

Mitigation category	Mitigation type	Representative citation
Drug	Caffeine	Penetar et al., 1993; Lieberman et al., 2002; Barry et al., 2005; Wesensten et al., 2005; Kohler et al., 2006; Killgore et al., 2011; McIntire et al., 2014; Paech et al., 2016; McIntire et al., 2017; Crooks et al., 2019; Killgore and Kamimori, 2020a
	Modafinil	Pigeau et al., 1995; Caldwell et al., 2000, Caldwell et al., 2004b; Wesensten et al., 2005
	D-amphetamine	Newhouse et al., 1989; Pigeau et al., 1995; Wesensten et al., 2005
	Donepezil	Dodds et al., 2011
	CX717 (AMPAKINE compound)	Boyle et al., 2012
Non-invasive stimulation	Transcranial direct current stimulation (tDCS)	Nitsche and Paulus, 2001; McIntire et al., 2014, 2017; Cheng et al., 2021
	Transcutaneous vagal nerve stimulation (tVNS)	Rizzo et al., 2003; McIntire et al., 2021
Other	Break period	Pigeau et al., 1995

### Mitigating cognitive effects

Caffeine has demonstrated consistent mitigating effects across many cognitive domains that are sensitive to sleep deprivation. For instance, caffeine leads to cognitive improvements in psychomotor vigilance speed, objectively measured alertness, visual vigilance, and performance on executive function tasks (Lieberman et al., 2002; Wesensten et al., 2005; Kohler et al., 2006; Paech et al., 2016). More specifically, caffeine administration has shown improvements in latencies on short-term memory tasks and has led to faster reaction times on psychomotor tasks (McIntire et al., 2014). Interestingly, the time of caffeine administration matters. McIntire et al. (2017) administered caffeine at one of two different times throughout the night. They found that the earlier the administration of caffeine, the greater the effects on reaction time speed. More specifically, it has been found that 2–4 h post-administration seems to be the optimal time for the effects of caffeine to fully kick in with the full effects lasting up to 6 h post-administration (Wesensten et al., 2005). Others have found that it only takes 1 h for caffeine to have its greatest effects and lasts for 8 h (Lieberman et al., 2002). Additionally, caffeine has prevented an increase in the number of risks taken when compared to placebo during sleep deprivation (Killgore et al., 2011). Caffeine also results in faster overall speed of PVT performance, a reduction in attentional lapses, a reduction in responsive lapses, and increases in novel problem-solving. Killgore and Kamimori (2020a) recently demonstrated this by testing the effectiveness of multiple doses of caffeine on several cognitive abilities. Twenty-three participants received 4 doses of caffeine or placebo during 77 h of sleep deprivation and completed a number of tests. Caffeine was indeed more effective than placebo across all tasks; however, the magnitude of these benefits did not restore abilities to baseline levels and the effects did not last after the first night.

Other substances have also been compared to the well-documented effects of caffeine. For instance, Wesensten et al. (2005) tested the efficacy of modafinil and d-amphetamine, two substances used for treatment of daytime sleepiness and narcolepsy, on performance and alertness. They found similar improvements on performance, alertness, psychomotor vigilance speed, and objectively measured alertness after administration of modafinil and d-amphetamine compared to caffeine. Additionally, they found that modafinil leads to an improvement on executive function tasks (i.e., improvements on the Biber Cognitive Estimation Performance) similar to the effects of caffeine. Conversely, they found that d-amphetamine leads to a decrease in performance on the Stroop task, demonstrating impairments in executive functioning. Furthermore, Pigeau et al. (1995) found that modafinil and d-amphetamine led to better performance on tasks involving reaction time, logical reasoning, and short-term memory compared to placebo. They also found that there was still a slight decline in performance compared to baseline (5–10% decline). Furthermore, they found that after a second administration of the drug, there was an even further decline in performance from baseline (20–30%) but still better performance than placebo. Additionally, Caldwell et al. (2004b) found that performance levels on a flight simulator decreased only slightly compared to baseline in the modafinil condition, but decreased significantly in the placebo condition. Boyle et al. (2012) utilized different doses of the drug CX717 in their study. They found that higher doses, CX717 may improve some tasks, but not all. For instance, they found that 1,000 mg of CX717 led to increases in performance on some attention-based tasks (i.e., reduction in the number of attentional slips on the Sustained Attention to Response Task, improved performance on psychomotor speed tests, and improved performance on the Critical Flicker Fusion test). On the other hand, they found that CX717 did not have an effect on memory, sensorimotor tasks, or alertness as assessed by multiple tests of alertness (i.e., Maintenance of Wakefulness Test, Continuous Tracking Task, Sustained Attention to Response Task, Rapid Visual Information Processing Task, and Critical Flicker Fusion). On the other hand, the substance donepezil failed to produce any significant effects on cognitive performance (Dodds et al., 2011).

Non-invasive stimulation is another well-documented technique to counteract fatigue. It has been shown that tDCS prevents a decrease in vigilance, leads to improvements in latencies on short-term memory tasks, leads to a faster reaction time on psychomotor tasks, increases target detection accuracy, and leads to improvements on PVT (McIntire et al., 2014, 2017). Furthermore, tDCS leads to improvements on attention, memory, and executive functioning compared to sham (Cheng et al., 2021). Recent studies have also worked on stimulating the Vagus nerve (i.e., tVNS). tVNS leads to increases in arousal, executive attention, and multitasking with participants performing better on PVT and Multi-Attribute Test Battery (MATB; McIntire et al., 2021). Additionally, tVNS increases alertness and decreases daytime sleepiness (Rizzo et al., 2003).

### Mitigating behavioral effects

The different mitigation strategies significantly affect behavior and mood states following a period of sleep deprivation. Caffeine has been shown to positively affect mood states (i.e., induce feelings of happiness, joy, and excitement), even at low doses (Lieberman et al., 2002). Furthermore, caffeine has been shown to reverse the effects of sleep deprivation on the Profile of Mood Scale (POMS) and VAS, thus improving mood (Penetar et al., 1993). Modafinil and d-amphetamine have also been shown to have a positive effect on mood states compared to placebo, but mood was slightly lower than the baseline mood state and steadily declined after initial increase (Pigeau et al., 1995). The dose of the drug determines the strength of the effect of the reversal of sleep deprivation effects on POMS and thus determines the strength of the increase in mood (Newhouse et al., 1989). However, the more sleep-deprived an individual is, the less effective mitigation drugs will be on increasing mood (Pigeau et al., 1995). Boyle et al. (2012) found that CX717 did not affect mood, even at high dose concentrations.

Non-invasive stimulation has also shown behavioral mitigating properties. McIntire et al. (2014, 2017) found that tDCS improves mood and decreases the negative effects associated with mood when stressors such as fatigue are involved. They found that changes in accuracy on various tasks were not correlated with mood changes, unlike caffeine. Furthermore, they concluded that tDCS may be more beneficial at mitigating fatigue with longer-lasting effects on mood. tVNS has also been shown to increase mood and energy levels (McIntire et al., 2021).

### Mitigating physiological effects

Although not necessarily the main goal for mitigating the effects of fatigue, changes in physiology are inevitable. For instance, an increase in HRV, decrease in heart rate, an unchanged pre Ejection period (PEP), and an overall increase in parasympathetic activity were observed following caffeine administration (Kohler et al., 2006; Crooks et al., 2019). Furthermore, it was found that caffeine resulted in an increase in skin conductivity, EEG alpha frequency, and a decrease in EEG alpha power (Barry et al., 2005). Other drugs, such as modafinil and d-amphetamine, have also been shown to have physiological effects and resulted in an increase in body temperature (Pigeau et al., 1995), a “pounding and racing” heartbeat (Wesensten et al., 2005), and a decrease in slow wave EEG activity (Caldwell et al., 2000). However, these results are not exclusive to the effects of caffeine on a sleep-deprived individual as other studies have shown similar physiological results of caffeine on those not involved in a sleep-deprivation paradigm (Richardson et al., 2004; Yeragani et al., 2005).

Even though the goal of caffeine is not to mitigate physiological effects, but rather mitigate feelings of sleepiness, the resulting physiological effects of mitigation techniques can be used to predict performance on complex tasks. Kohler et al. (2006) found that HRV and HR were useful at predicting response time and accuracy, with and without caffeine administration, on grammatical reasoning tasks. They determined that tasks that require higher cognitive processing are affected by increases in parasympathetic activity (i.e., caused by caffeine).

### Mitigating subjective effects

Mitigation techniques have consistently shown to have their effects on subjective measures. Caffeine leads to a decrease in self-reported risk levels (Killgore et al., 2011), decrease in subjective sleepiness (Wesensten et al., 2005), lower fatigue ratings (McIntire et al., 2017), and fewer self-reported negative side effects (Killgore and Kamimori, 2020a). Furthermore, Lieberman et al. (2002) found that 100 mg of caffeine was not enough to have mitigating effects on subjective sleepiness, but 200 and 300 mg were. Additionally, it has been found that modafinil and d-amphetamine also led to participants feeling less sleepy, less fatigued, and less depressed compared to placebo (Pigeau et al., 1995; Caldwell et al., 2004b; Wesensten et al., 2005). Furthermore, donepezil has been found to reduce feelings of sleepiness and increase feelings of alertness (Dodds et al., 2011).

Non-invasive stimulation has also been found to have effects on subjective measures. McIntire et al. (2014, 2017) found that after tDCS, participants subjectively felt less fatigued, less drowsy, had more energy, and felt like they were in a better mood state (i.e., felt more happy and optimistic) compared to both placebo and caffeine conditions. Furthermore, they found that tDCS led to participants feeling more confident in their ability to do a task and fewer feelings of boredom. Similarly, participants reported lower feelings of fatigue and felt like they had more energy compared to sham following tVNS.

## Discussion

As aforementioned, fatigue due to sleep deprivation affects many aspects of human performance. Performance deficits are found in cognitive, behavioral, physiological, and subjective domains. There have been many efforts to identify predictors and countermeasures for fatigue due to sleep deprivation in order to mitigate its effects. However, due to individual differences, and the fact that some indicators of fatigue are not solely indicative of being in a fatigued state (i.e., increased response times may indicate boredom, cognitive load, mind-wandering, etc.), we propose that a multimodal system that records and analyzes multiple sources of physiological and biological response data in real-time may provide an informative model of fatigue-related performance effects and provide individualized mitigation techniques.

Prior work has suggested this notion as well, based on the relationships between sleep deprivation and measures such as oculometrics (Goldich et al., 2010; Abe et al., 2011; Ftouni et al., 2013; Jackson et al., 2016), sleep history (Mantua et al., 2021), and physiology (Chua et al., 2012; Glos et al., 2014). For instance, Mantua et al. (2021) studied the impact of subjective sleep need and sleep resilience on performance. They found that subjective sleep and sleep resilience are insufficient at predicting performance individually, however, their interaction can predict performance effects more accurately. Some ocular metrics (i.e., PERCLOS) have been found to parallel changes in subjective sleepiness, performance decrements, and are sensitive to sleep deprivation (Abe et al., 2011; Ftouni et al., 2013; Jackson et al., 2016). For instance, Ftouni et al. (2013) found that ocular metrics such as the Johns Drowsiness scale, blink duration, and percentage of time with eyes closed displayed strong temporal relationships with subjective sleepiness and attentional performance on the PVT. Similarly, Chua et al. (2012) found that monitoring HRV also provides insight into when individuals are approaching fatigue; specifically, they found that HRV performed as well as PERCLOS in predicting attentional lapses on the PVT. This leads us to posit the idea of a closed-loop system that would assess fatigue, mitigate fatigue, and monitor the outcome of those mitigations (physiologically), all in real-time. Prior work in similar fields has found the use of closed-loop systems to be useful at monitoring and predicting performance deficits in real-time (Wilson et al., 2007; Christensen and Estepp, 2013).

An important component of closed-loop systems is the ability to monitor the effects of fatigue on performance in real-time. The length of effect of some mitigation strategies has been well documented. For instance, the half-life of caffeine has been observed to be around 4–6 h depending on the dose (Penetar et al., 1993; Wesensten et al., 2005; Killgore et al., 2006a). Even though the half-life of caffeine is well documented, individuals have different tolerance levels to caffeine. For instance, it has been observed that individuals who don’t regularly drink caffeine experience a longer half-life following consumption compared to caffeine drinkers (5.3 h compared to 4.1 h; Whitsett et al., 1984). Furthermore, non-users experience an increase in physical performance following caffeine administration compared to users (Bell and McLellan, 2002). Although the length of effect of caffeine is well known, the length of effects of other mitigation techniques is not. For instance, the duration of effects of newer methods such as tVNS and tDCS are less clear. Some have found the effects of tDCS to last up to 24 h (McIntire et al., 2017), whereas others report effects lasting 30–90 min (Nitsche and Paulus, 2001). Furthermore, the duration of tDCS effects has been found to depend on the duration of the stimulation session as well as on the number of times a participant is stimulated (McKinley et al., 2012). Although the duration of effects for tVNS and tDCS is less clear, we propose that real-time assessment would be useful in order to monitor the duration, intensity, and effectiveness of each mitigation to optimize performance for each individual.

Furthermore, we propose that multimodal systems, monitored in real-time, will be beneficial in determining optimal mitigation strategies since individuals have different resistance levels to fatigue. For instance, Van Dongen et al. (2004) found significant differences in levels of subjective sleepiness and mood, cognitive processing ability, and alertness among individuals. They found that these results were not affected by sleep history (i.e., how much sleep they got before testing), but rather by some other trait. Furthermore, Mantua et al. (2021) studied the effects of individual need for sleep on the PVT. They found that individuals who were less resistant to fatigue performed worse on the PVT compared to those with medium to high resistance. Philip et al. (2004) studied the impact of age on performance. They found that younger participants were not as proficient in gauging their level of fatigue compared to older participants. Reaction times between the groups were similar, but perception of performance was not. Lastly, some neuroimaging studies have identified that individual differences in brain structures and activation patterns during sleep deprivation may be able to predict their vulnerability to fatigue (Mu et al., 2005; Rocklage et al., 2009). Since each individual perceives and reacts differently to fatigue, it is nearly impossible to have a single mitigation strategy to counteract fatigue, further proving the need for a real-time, individualized system.

## Conclusion

Sleep deprivation due to fatigue leads to a wide range of performance decrements that may pose occupational risks. Many studies have looked at the effects of sleep deprivation on cognitive, physiological, behavioral, and subjective measures. For example, recent experiments reveal that a night of no sleep leads to a decreased ability to respond to tasks properly resulting in slower response times, increased lapses in attention, and declines in memory performance. Furthermore, other experiments have shown that sleep deprivation leads to increased subjective sleepiness and an increase in behavioral outcomes such that participants experience an increase in risk-taking, decrease in inhibitory controls, decreased ability to detect and correct errors, and an increased likelihood to make mistakes. Additionally, studies have also shown physiological effects resulting from fatigue such that there is an increase in sympathetic activity and a decrease in parasympathetic activity. Many studies have utilized different mitigation strategies to attempt to counteract these deleterious effects of not getting enough sleep. For example, many studies have utilized different drugs, such as caffeine and modafinil whereas other studies have used non-invasive stimulation to counteract these effects. Although these mitigation techniques have proven to be useful, individual differences make it difficult to determine when to apply the intervention to optimize its potential and minimize the effects of sleep deprivation. Further research studying closed-loop mitigation systems in sleep deprivation is needed to determine the best time and techniques for each individual. In addition, there is also value in exploring unique methods and techniques not reviewed here (with appropriate theoretical justification), as their absence from this review may suggest they are either unknown or under-studied, to date.

## Author contributions

KK, VP, and JE defined the scope of the review, outlined the manuscript, and edited the manuscript. KK and VP performed the literature and drafted the primary manuscript. KK created and edited the summary tables. All authors contributed to the article and approved the submitted version.

## Author disclaimer

The views expressed are those of the authors and do not necessarily reflect the official policy or position of the Department of the Air Force, the Department of Defense, or the United States Government. The appearance of external hyperlinks does not constitute endorsement by the United States Department of Defense of the linked websites or the information, products, or services contained therein. The Department of Defense does not exercise any editorial, security, or other control over the information you may find at these location.
