# Supplementary material for: Predicting and mitigating fatigue effects due to sleep deprivation: A review
Source: Front Neurosci. 2022 Aug 5;16:930280. doi: 10.3389/fnins.2022.930280 (PMC9389006; doi:10.3389/fnins.2022.930280)
Supplement: Supplementary file 1 [file Table_1.docx]

**Supplementary Table 1.** Summary of the types of measurements for different types of effects of fatigue on cognition.

| **Fatigue related variations in performance** | **Domain** | **Sub-Domain** | **Effect** | **Measurements** | **Representative Citation** |
| --- | --- | --- | --- | --- | --- |
| Effects on cognition | Inhibitory control |  | Decrease in inhibitory control | Go/no go task | Zhong et al. (2005); Chuah et al. (2006); Drummond et al. (2006) |
|  |  |  |  | Bimodal Stroop Task | Deliens et al. (2018) |
|  |  |  |  | Inhibition Stop Signal task | Slama et al. (2018) |
|  | Risk taking |  | Increase in risk taking | Balloon Analog Risk Task | Killgore et al. (2011) |
|  |  |  |  | Iowa Gambling Task | Killgore et al. (2006a) |
|  |  |  |  | Risk Taking Assessment | Choshen-Hillel et al. (2021) |
|  | Reaction time/lapses | Reaction time | Increase in reaction time | Psychomotor Vigilance Test (PVT) | Neri et al. (2002); Van Dongen et al. (2003b); Van Dongen et al. (2004); Wesensten et al. (2005); Kohler et al. (2006); Wilson et al. (2007); Franzen et al. (2008); Doran et al. (2001); Mollicone et al. (2010); McKinley et al. (2011); Chua et al. (2012); Gorgoni et al. (2014); McIntire et al. (2014 and 2017); Paech et al. (2016); Jackson et al. (2016); Boardman et al. (2018); McMahon et al. (2018); Slama et al. (2018); Killgore and Kamimori (2020a); Sun et al. (2020); Cheng et al. (2021) |
|  |  |  |  | Simple Reaction Time Test | Philip et al. (2004); Zhong et al. (2005); Philip et al. (2012) |
|  |  |  |  | Brief Stimulus Reaction Test | Romeijn et al. (2012) |
|  |  |  |  | Four Choice Visual Reaction Time Test | Lieberman et al. (2002) |
|  |  |  |  | Choice Reaction Time Task | Orton and Gruzelier (1989) |
|  |  |  |  | Vigilance Reaction Time Task | Orton and Gruzelier (1989) |
|  |  |  |  | Serial Reaction Time Task | Pigeau et al. (1995); Axelsson et al. (2008) |
|  |  |  |  | Ruler Dropping Test | Patrick et al. (2017) |
|  |  |  |  | Auditory reaction time test | Ogilvie and Wilkinson (1984) |
|  |  |  |  | Responding to stimuli | Bartel et al. (2004) |
|  |  | Lapses | Increase in lapses | PVT | Dinges et al. (1997); Howard et al. (2003); Van Dongen et al. (2003b); Chuah et al. (2006); Kohler et al. (2006); Wilson et al. (2007); Anderson and Dickinson (2010); Banks et al. (2010); Mollicone et al. (2010); McKinley et al. (2011); Chua et al. (2012); Buckley et al. (2016); Paech et al. (2016); Jackson et al. (2016); Lo et al. (2016); McMahon et al. (2018); Mantua et al. (2021); Sun et al. (2020); Killgore et al. (2020b) |
|  |  |  |  | Brief Stimulus Reaction Task | Romeijn et al. (2012) |
|  |  |  |  | 2D Tracking Task | Buckley et al. (2016) |
|  | Decision making/ constructive thinking | Decision making | Decision making is impaired | Iowa Gambling Task | Killgore et al. (2006a) |
|  |  |  |  | Balloon Analog Risk Task | Killgore et al. (2011) |
|  |  |  |  | Task Goal Switching Task | Slama et al. (2018); |
|  |  |  |  | Visual Motor Task | Rocklage et al. (2009) |
|  |  |  |  | Task Switching Paradigm | Bratzke et al. (2009); Couyoumdjian et al. (2010) |
|  |  |  |  | Flexibility task | Deliens et al. (2018) |
|  |  | Constructive thinking | Impaired constructive thinking | Constructive Thinking Inventory | Killgore et al. (2008) |
|  |  |  |  | The Tower of Hanoi | Killgore and Kamimori (2020a) |
|  |  |  |  | Logical Reasoning Task | Pigeau et al. (1995) |
|  |  | Flexible thought processing | Decrease in flexible thought processing | Flexibility task | Deliens et al. (2018) |
|  | Memory | Short Term Memory | Decrease in short term memory | Sternberg Short Term Memory Scanning Task | Boyle et al. (2012) |
|  |  |  |  | Repeated Acquisition Test | Lieberman et al. (2002) |
|  |  |  |  | Sequential Reaction Time Memory Task | Bartel et al. (2004) |
|  |  |  |  | Digit Span Task | Pigeau et al. (1995) |
|  |  | Working Memory | Decline in working memory | N-Back Task | Dodds et al. (2011); Deliens et al. (2018); Gerhardsson et al. (2019); Slama et al. (2018) |
|  |  |  |  | Digit Symbol Substitution Test | VanDongen et al. (2003b and 2004) |
|  |  |  |  | Immediate and Delayed Word Recall | Boyle et al. (2012) |
|  |  |  |  | Delayed Matching-to-Sample Task | McIntire et al. (2014 and 2017) |
|  |  |  |  | Cogstate Brief Battery | McMahon et al. (2018) |
|  |  |  |  | One Card Learning Task | McMahon et al. (2018) |
|  |  |  |  | One Back Task | McMahon et al. (2018) |
|  |  |  |  | Sternberg Working Memory Task | Mu et al. (2005) |
|  |  |  |  | The SIMON game | Patrick et al. (2017) |
|  |  |  |  | Pattern Recognition Task | Dodds et al. (2011) |
|  |  |  |  | Arithmetic task | Drummond et al. (1999); Van Dongen et al. (2003b and 2004); Minkel et al. (2012); Boardman et al. (2018) |
|  |  |  |  | Matching to Sample Task | Lieberman et al. (2002) |
|  |  |  |  | Sequential Reaction Task | Bartel et al. (2004) |
|  |  |  |  | Delayed Recognition Span Test | Rollinson et al. (2003) |
|  |  | Memory Recall | Decrease in memory recall ability | Misinformation Paradigm | Lo et al. (2016) |
|  |  |  |  | Brief Visuospatial Memory Test | Cheng et al. (2021) |
|  |  |  |  | Probed Recall Memory | Howard et al. (2003) |
|  | Attention/ vigilance |  | Decline in attention/ vigilance | Mackworth Clock Test | McIntire et al. (2014 and 2017) |
|  |  |  |  | Modified Maintenance of Wakefulness Test | Boyle et al. (2012) |
|  |  |  |  | Oxford sleep resistance test | Wilkinson et al. (2013) |
|  |  |  |  | Fatigue Symptoms Questionnaire | Bourgeois-Bougrine et al. (2003) |
|  |  |  |  | Continuous Tracking task | Boyle et al. (2012) |
|  |  |  |  | Tracking task | Kohler et al. (2006) |
|  |  |  |  | Electrooculography (EOG)/ slow eye movements | Neri et al. (2002); Lockley et al. (2004) |
|  |  |  |  | Sustained Attention to Response Task | Boyle et al. (2012) |
|  |  |  |  | Rapid Visual Information Processing Task | Boyle et al. (2012) |
|  |  |  |  | Continuous Performance Test | Wu et al. (1991); Rollinson et al. (2003) |
|  |  |  |  | Visual Vigilance Task | Wu et al. (1991) |
|  |  |  |  | MOXO-Continuous Performance Test | Choshen-Hillel et al. (2021) |
|  |  |  |  | Scanning Visual Vigilance Test | Lieberman et al. (2002) |
|  |  |  |  | Digit Cancellation test | Cheng et al. (2021) |
|  |  |  |  | Test for attentional performance | Deliens et al. (2018) |
|  |  |  |  | PVT | Dinges et al. (1997); Doran et al. (2001); Neri et al. (2002); Howard et al. (2003); Van Dongen et al. (2003b); Van Dongen et al. (2004); Kohler et al. (2006); Wilson et al. (2007); Franzen et al. (2008); Previc et al. (2009); Anderson and Dickinson (2010); Wilkinson et al. (2013); Gorgoni et al. (2014); McIntire et al. (2014 and 2017); Buckley et al. (2016); Jackson et al. (2016); Lo et al. (2016); Deliens et al. (2018); Slama et al. (2018); Abe et al. (2020); Sun et al. (2020) |
|  |  |  |  | Auditory Psychomotor Vigilance Task | Ftouni et al. (2013) |
|  |  |  |  | Identification task | McMahon et al. (2018) |
|  |  |  |  | Detection task | McMahon et al. (2018) |
|  |  |  |  | 2D tracking task | Buckley et al. (2016) |
|  |  |  |  | Auditory Go/no go task | Chua et al. (2017) |
|  |  |  |  | Visual go/no go task | Chua et al. (2017) |
|  |  |  |  | Motor tracking task | Chua et al. (2017) |
|  |  |  |  | Karolinski Drowsiness Test | Ftouni et al. (2013); Glos et al. (2014) |
|  |  |  |  | Maintenance of Wakefulness test | Wesensten et al. (2005) |
|  |  |  |  | Brief Stimulus Reaction Task | Romeijn et al. (2012) |
|  | Executive Function |  | Decline in Executive function | Cambridge Neuropsychological Test Automated Battery | Dodds et al. (2011) |
|  |  |  |  | Controlled Oral Word Association | Wesensten et al. (2005) |
|  |  |  |  | Animal fluency | Wesensten et al. (2005) |
|  |  |  |  | Wisconsin card sorting task | Wesensten et al. (2005) |
|  |  |  |  | Stroop test | Wesensten et al. (2005); Minkel et al. (2012); Michael et al. (2013); Patrick et al. (2017); Deliens et al. (2018); Cheng et al. (2021) |
|  |  |  |  | Trail Making tests | Cheng et al. (2021) |
|  |  |  |  | Biber cognitive estimation test | Wesensten et al. (2005) |
|  |  |  |  | The Behavior Rating Inventory of Executive Function-Adult Version | Choshen-Hillel et al. (2021) |
|  | Error detection/ correction |  | Decline in ability to detect/correct errors | Letter Flanker | Hsieh et al. (2007) |
|  | Cognitive Performance/ Function |  | Decrease in cognitive performance and function | Multi-Attribute Test Battery (MATB) | Wilson et al. (2007); Previc et al. (2009) |
|  |  |  |  | Operation Span Task | Previc et al. (2009) |
|  |  |  |  | Word Detection task | Van Dongen et al. (2004) |
|  |  |  |  | Operator Vehicle Interface Task | Wilson et al. (2007) |
|  |  |  |  | Wechsler Adult Intelligence Scale | Van Dongen et al. (2004) |
|  |  |  |  | Target identification tasks | McKinley et al. (2011) |
|  |  |  |  | Haptic sorting tests | Orton and Gruzelier (1989) |
|  |  |  |  | Verbal Learning task | Drummond et al. (2000); Halbach et al. (2003); Drummond et al. (2005) |
|  |  |  |  | Warship commander task | McIntire et al. (2017) |
|  |  |  |  | Critical Flicker Fusion Test | Boyle et al. (2012) |
|  |  |  |  | Digit Symbol Substitution Test | Boyle et al. (2012); Paech et al. (2016) |
|  |  |  |  | Grammatical reasoning task | Kohler et al. (2006) |
|  |  |  |  | Haylings sentence completion task | Harrison and Horne (1998) |
|  |  |  |  | Word generation task | Harrison and Horne (1998) |
